# Methylphenidate treatment for cognitive symptoms associated with ADHD in a pediatric epilepsy patient following resection of a left frontal cortical dysplasia

**DOI:** 10.1016/j.ebr.2021.100435

**Published:** 2021-02-10

**Authors:** Donald J. Bearden, Sidra Shakil, David O'Banion, Kim E. Ono, Daniel L. Drane, David W. Loring, Daniel C. Tarquinio

**Affiliations:** a Department of Neuropsychology, Children’s Healthcare of Atlanta, Atlanta, GA, USA; b Department of Neurology, Emory University School of Medicine, Atlanta, GA, USA; c Alabama College of Osteopathic Medicine, Dothan, AL, USA; d Department of Pediatrics, Emory University School of Medicine, Atlanta, GA, USA; e Department of Developmental Pediatrics, Children’s Healthcare of Atlanta, Atlanta, GA, USA; f Department of Neurology University of Washington School of Medicine, Seattle, WA, USA; g Center for Rare Neurological Diseases, Atlanta, GA, USA

**Keywords:** Pediatric epilepsy, Epilepsy surgery, Left frontal cortical dysplasia, Attention deficit hyperactivity disorder, Methylphenidate

## Abstract

We present data on a 10-year-old patient with drug-resistant epilepsy who was treated with methylphenidate for symptoms of attention deficit hyperactivity disorder (ADHD) that developed after she underwent surgical resection of a left frontal cortical dysplasia. . The patient’s parents reported methylphenidate was helpful in improving their child’s reading performance. Based on parents’ report, we examined benefits of methylphenidate on our patient’s cognitive problems in a controlled setting. The patient underwent a neuropsychological evaluation completed in three sessions over a five-day period. Methylphenidate was administered prior to the second testing session only and was associated with improvements in the patient’s attention, executive function, processing speed, and short-term memory performances. In comparison, word-reading performance, a task less susceptible to neurological impairment, was stable over the three sessions. The patient remained seizure-free after surgery and use of methylphenidate did not reduce seizure threshold. These findings support the use of methylphenidate in treating targeted cognitive problems associated with ADHD emerging after epilepsy surgery in children.

## Highlights

• ADHD symptoms can emerge or worsen after epilepsy surgery.
• Methylphenidate use improved cognitive symptoms of ADHD in our patient.
• Seizure aggravation did not occur following use of a neurostimulant in our patient.
• Our patient benefitted from a multidisciplinary intervention approach.

## 1 Introduction.

Attention deficit hyperactivity disorder (ADHD) affects up to a third of children with epilepsy [1], [2], [3], [4]. Young people with comorbid epilepsy and ADHD exhibit more severe cognitive difficulties than their peers with ADHD alone [5], [6]. Frontal lobe epilepsy is the second most common form of focal epilepsy [7]. One study indicated that pediatric patients with frontal lobe epilepsy are at higher risk for being diagnosed with ADHD than patients with other epilepsy types [7]; however, other studies have not supported this finding [4], [8], [9], [10], [11]. Patients with frontal lobe epilepsy and current abnormal EEG discharges are at higher risk of having ADHD than those without EEG discharges, either due to transient frontal lobe dysfunction resulting from interictal abnormalities or frank cortical injury [12]. Within ADHD population studies, evidence suggests that children with epilepsy are more commonly diagnosed with predominantly inattentive type [4], [8], [13], which often includes problems with attention, executive function, information processing speed, and short-term memory. Further, cognitive difficulties including those associated with ADHD may be exacerbated by epilepsy-related sequelae, including commonly prescribed antiseizure medications (ASMs) [14] and sleep difficulties [15].

Following pediatric epilepsy surgery, ADHD symptoms may remain or emerge de novo [8]. Among pediatric epilepsy surgical patients, researchers found attention problems to be more common in children undergoing frontal lobe resections than those undergoing temporal or multi-lobar excisions [16], [17], although attention deficits can emerge as sequelae of nearly any surgical brain procedure. Despite the elevated frequency of attention problems in pediatric patients following epilepsy surgery, few data exist to support or refute the safety and efficacy of stimulant medication after surgery [8].

Methylphenidate is a frontline treatment for ADHD, is safe and effective for patients with epilepsy [18] and can contribute to improvements in their quality of life [19], [20]. However, use of methylphenidate in the context of epilepsy is not helpful for everyone and may increase seizure frequency, particularly at higher dosages [19], [21], [22], and may worsen EEG findings [23]. Therefore, these patients require close monitoring, especially if seizures are not well controlled and/or there is a history of status epilepticus or frequent generalized seizures. Our pediatric case study highlights the potential benefits of using methylphenidate to manage common ADHD symptoms that can develop following epilepsy surgery.

## 2 Methods.

We conducted a post-surgical neuropsychological evaluation with our patient approximately one-year following epilepsy surgery to examine the effectiveness of methylphenidate on targeted ADHD symptoms. The evaluation was conducted in three sessions over a five-day period. The patient was administered methylphenidate during the second testing session only as part of an N-of-one trial with an A-B-A research design. Cognitive areas of interest were those commonly problematic in individuals with ADHD, including attention, executive function, processing speed, and short-term memory. As a point of comparison, we included an untimed word-reading task during each session because this skill is more stable and less susceptible to neurological injury/disease [24]. We compared the patient’s performances across the three testing sessions using standard deviation (SD) change as has been done in previous studies [25], [26]. Within this context, a change between one and two SDs was considered mild to moderate and a change of two SDs or greater was considered clinically significant. In addition, we reviewed the patient’s presurgical workup, surgical procedure, and outcome following surgery.

## 3 Results: Case presentation.

### 3.1 Patient demographics and disease-specific factors

Our patient was diagnosed with drug-resistant epilepsy at nine years of age. She was born following a 42-week, uncomplicated pregnancy weighing eight pounds. Maternal tobacco use was reported during pregnancy. No problems were reported with perinatal course. Developmental motor milestones were achieved within age-level expectations and speech/language skills were delayed. At age nine, the patient began experiencing frequent focal seizures with alteration of awareness that included staring, unresponsiveness, and hypermotor activity lasting 10–30 seconds. The frequency of the episodes increased acutely up to 40 times per day. Postictal features included amnesia for what she was doing immediately prior to the episode. No family history of seizures was reported. Past medical history included irritable bowel syndrome. Following the patient’s presentation of seizures, she was rapidly up-titrated on several ASMs including phenytoin, oxcarbazepine, and lamotrigine. None of these controlled her seizures; although frequency decreased somewhat, the seizures continued to occur daily.

### 3.2 Presurgical functional status

Our patient was in the third grade when she presented for presurgical evaluation. She had received special education services through an Individualized Education Plan under the category of speech/language impairment in pre-kindergarten and kindergarten, which provided her with speech/language therapy in school. She had not since received any formal academic support services. The patient’s mother reported that her daughter was an A-B student prior to seizure onset and began having more trouble at the start of her third-grade year. According to her most recent report card prior to surgery, the patient was earning Cs in all classes. Math was an area of long-standing difficulty. Mild concerns were reported by her teacher regarding inattention, but these were believed to be due to seizure activity during class.

### 3.3 Presurgical epilepsy evaluation

**Fig. 3:**
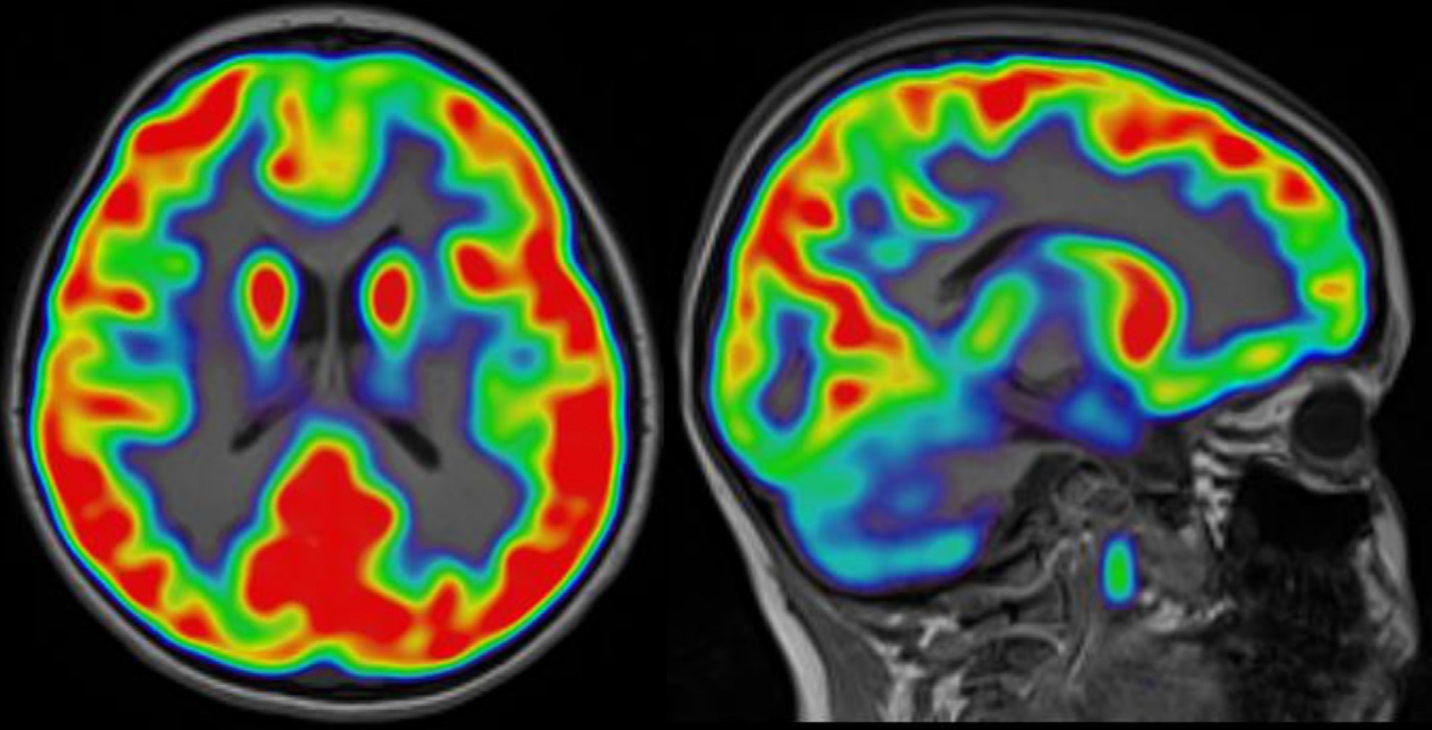
Fluorodeoxyglucose-positron emission tomography (PET) demonstrating hypometabolism in left frontal pole.

During the patient’s clinical evaluation, the electroencephalogram (EEG) demonstrated focal epileptiform discharges arising from the left frontal polar region (Fig. 1a, Fig. 1b). A three-Tesla brain magnetic resonance imaging (MRI) scan showed focal cortical dysplasia described as an area of non-enhancing, hyperintense signal in the cortex and underlying white matter in the anteromedial inferior left frontal lobe. Evidence of abnormal cortical thickening, blurring of the gray-white junction, and mild local mass effect was noted (Fig. 2a, Fig. 2b). Results of a positron emission tomography (PET) scan revealed an area of focal, subtle hypometabolism corresponding to the cortical dysplasia identified on MRI (Fig. 3). Single-photon emission computed tomography (SPECT) findings were non-contributory.

### 3.4 Presurgical neuropsychological evaluation results

The patient underwent a comprehensive neuropsychological evaluation as part of her presurgical workup. Findings from the assessment indicated that her intellectual functioning was in the low average range, with evenly developed verbal and nonverbal abilities. Her attention, processing speed, short-term memory, and word-reading skills were within the broad average range (Table 1).

### 3.5 Surgical procedure

The patient underwent surgical resection of the left frontal cortical dysplasia to address her seizures at age nine years old. Post-surgical pathology indicated type IIb cortical dysplasia. Following surgery, the patient was seizure-free (Engel’s Class I outcome) and was weaned off all ASMs three months following surgery.

A post-operative brain MRI revealed findings consistent with her resection, with a subjacent anterior medial area of encephalomalacia in the left frontal lobe and minimal subdural hemorrhage adjacent to this site (Fig. 4a, Fig. 4b). A postsurgical EEG was abnormal due to infrequent sharp-and-slow wave discharges seen over the left frontal region in addition to left frontal delta slowing under a breach rhythm.

### 3.6 Post-surgical outcome

During her most recent postsurgical neurological examination, the patient presented as alert and interactive with a normal level of activity for her age. Qualitatively, she exhibited normal verbal output, eye contact, and nonverbal interactions. Her physical examination was within normal limits for her age. Over the course of the examination, the patient’s parents reported major concerns about her attention and memory problems following surgery that were disrupting daily functioning. Attention problems reported by parents resulted in a diagnosis of ADHD, predominantly inattentive type, and her neurologist (DCT) prescribed methylphenidate (10 mg per day) and recommended neuropsychological testing. After starting methylphenidate, the patient’s parents reported dramatic improvements in her attention that resulted in her reading skill improving an entire grade level at school.

As a result of the patient’s dramatic improvement, we sought to empirically validate parents’ report with the current study. The study was a multidisciplinary and interdisciplinary collaboration that included the patient’s neurologist (DCT), a developmental pediatrician (DO), and neuropsychology team (DJB, KEO, DLD, & DWL). Together, we conducted an N-of-one trial to assess medication-related performance changes using an A-B-A research design.

### 3.7 Post-surgical neuropsychological evaluation

The patient underwent a comprehensive neuropsychological evaluation over three sessions within a consecutive five-day period approximately one year after surgical resection of the left frontal dysplastic area (age 10 years old). Session one established a baseline on tasks of relevance (i.e., attention, executive function, processing speed, and short-term memory), session two assessed the immediate treatment effects of methylphenidate, and session three reassessed the patient after discontinuing methylphenidate. Initial findings indicated the patient’s overall intellectual ability was within the low average range with equally developed verbal and nonverbal skills, consistent with presurgical evaluation findings.

To examine the effects of methylphenidate on the patient’s cognitive skills, multiple measures were used. She was assessed with the Conners’ Continuous Performance Test, Third Edition (CPT-3) [27]. The CPT-3 is a well-established, computerized test and considered a gold-standard tool for assessing sustained attention/executive function skills, including inattentiveness, impulsivity, and vigilance, though some studies have found limitations in earlier versions of the measure’s ability to predict behavior in real-world milieus [28], [29], [30]. One study [30], however, found that omission errors and variable response times clearly differentiated children with ADHD from healthy controls on the second edition Conners’ Continuous Performance Test (CPT-II) [31].

Processing speed was assessed with the Coding subtest of the Wechsler Intelligence Scale for Children, Fifth Edition (WISC-V) [32]. Coding is a timed task (120 seconds) that requires the examinee to draw a symbol that corresponds to a number as quickly as possible without making mistakes. According to Wechsler [32], the task assesses processing speed, as well as “short-term visual memory, procedural and incidental learning ability, psychomotor speed, visual perception, visual-motor coordination, visual scanning ability, cognitive flexibility, attention, concentration, and motivation.”

Due to the lack of available pediatric memory measures with alternative forms, and the likelihood of practice effects if the same memory measures are repeated over a short time period, short-term memory was examined using three distinct, but similar verbal memory measures. These included the Story Memory subtest from the Wide Range Assessment of Memory and Learning, Second Edition (WRAML-2) [33], Instructions subtest from the Child and Adolescent Memory Profile (ChAMP) [34], and Stories subtest from the Children’s Memory Scale (CMS) [35]. All three of these measures are commonly used in pediatric neuropsychological evaluations to assess immediate (i.e., short-term), contextualized verbal memory. In this context, immediate verbal memory is conceptualized as the ability to hold and organize auditory-verbal information in mind for a short period of time until it needs to be recalled [35].

As a point of comparison, we included a word-reading task during each test session (Woodcock-Johnson Tests of Achievement, 4th Edition (WJ-IV), Letter-Word Identification, Forms A & B) [36]. Word-reading tasks have been found to be more stable and less susceptible to neurological damage when compared to tasks that tap online, in-the-moment information processing skills such as processing speed and attention [24].

### 3.8 Observations & analyses

To assess for statistically significant changes in the patient’s performance on attention/executive function and processing speed tasks, we examined standard deviation change in the patient’s test performance among the three evaluation sessions as has been done in prior research [25], [26]. A one-standard deviation change was interpreted as mild to moderate, and a two-standard deviation or greater change was interpreted as significant. Standard deviation change in performance could not be calculated for short-term verbal memory because different verbal memory measures were used. Thus, findings in this domain are discussed qualitatively.

Examiner observations of the patient over the testing sessions included increased distractibility and fidgetiness when not medicated (sessions one and three) compared to when medicated (session two). Otherwise, the patient was motivated to perform well and put forth good effort. There was no indication of validity issues; results were considered valid.

Analyses of test data indicated improvements of relative to significant magnitude in the patient’s performance associated with methylphenidate use (Table 2, Table 3, Table 4) across all experimental tasks. On the CPT-3 (Table 2), improvements were seen in detectability (i.e., ability to differentiate between targets and non-targets), number of missed targets (i.e., omission errors), perseveration errors, hit reaction time (i.e., response speed), response speed inconsistency (i.e., hit reaction time standard deviation), and variability of response speeds across sub-blocks of the test (i.e., variability). A similar trend was seen in the patient’s processing speed skill (WISC-V Coding) (Table 3); however, the difference was only significant between sessions two and three.

Qualitative comparison of the patient’s performance on three different verbal memory tasks (i.e., WRAML-2 Stories; ChAMP Instructions; CMS Stories) (Table 4) suggested that her short-term memory improved when she was treated with methylphenidate compared to when she was not. Compared to the patient’s performance across the three trials on the experimental tasks, her performance on a word-reading task (WJ-IV Letter-Word Identification) remained stable over the three test sessions (Table 5).

## 4 Discussion.

Overall, neuropsychological findings from our study suggest a positive effect of methylphenidate on performance in areas of sustained attention, executive function, processing speed, and short-term verbal memory, which is consistent with findings from previous studies indicating that methylphenidate improves performance in these areas in individuals with epilepsy [22], [37]. In comparison, methylphenidate did not affect performance on a word-reading task, a finding that is in line with research indicating that this skill is more stable than skills of interest in our study [24]. Our patient was only prescribed 10 mg of methylphenidate, supporting prior research showing that low to moderate doses of methylphenidate are safe and effective at reducing ADHD symptoms and improving quality of life in pediatric patients with difficult-to-treat-epilepsies [18], [38], [39], [40]. Furthermore, our findings indicated methylphenidate can be beneficial for patients following epilepsy surgery despite continued abnormal EEG findings. Nevertheless, methylphenidate use may increase seizure frequency [19], [21], [22], [41], [42], [43] and worsen EEG results [23]. Therefore, we recommend closely monitoring these patients when prescribing methylphenidate or other stimulants.

An additional reason for closely monitoring pediatric epilepsy patients treated with methylphenidate is the myriad factors that may contribute to problems in these individuals, which differ when compared to those seen in children with ADHD alone. For example, ictal and interictal epileptiform discharges alike can contribute to altered attentional processing [44], [45]. Certain ASM’s, such as benzodiazepines and decarboxylase inhibitors can affect attention as well [46], [47]. Sleep problems are a common comorbidity in patients with epilepsy [15] and can result in difficulty with alertness and attention. As in the current case, attention problems can appear or worsen after undergoing neurosurgical intervention [48], [49]. Therefore, prior to prescribing methylphenidate a comprehensive neurological examination is necessary, including EEG and a thorough behavioral analysis.

In general, treatment of pediatric ADHD should follow established guidelines [50], which consist of monitoring the effect of stimulant medications on the changes in core ADHD symptoms. Following this approach, clinicians should gather repeated rating scales of behavior while their patients take stimulants. This can be an onerous task, and most often is not completed [51], which leaves medication titration essentially unmeasured.

A unique aspect of our study is that we were able to empirically validate parents’ subjective report of methylphenidate-related improvements in their child’s reading ability with respect to attention. Children with epilepsy who have ADHD often improve [43] on stimulant medication, but rarely report such a specific improvement as the one seen here. This may be due to the location of our patient’s dysplasia within the frontal lobe and the role this brain region plays in attention during reading. Additionally, our study demonstrated that methylphenidate can be used to successfully treat cognitive problems associated with ADHD in pediatric patients following epilepsy surgery without lowering seizure threshold. Another strength of the current study is that it supports the use of multidisciplinary and interdisciplinary team approaches when treating patients diagnosed with epilepsy, especially more complex patients with comorbid diagnoses. Specifically, our patient’s care required the expertise of a pediatric neurologist, a developmental pediatrician, and neuropsychologists.

Our study also has limitations. First, it examines a single subject, which limits generalizability of findings. Next, practice effects may have played a role in our findings on sustained attention/executive function and processing speed tasks. However, despite the possible confounding influence of practice effects, the patient’s attention, executive function, and processing speed performances declined on the third evaluation session, adding further support to the effectiveness of methylphenidate in improving these skills. Another weakness of our study pertains to the use of three different verbal memory measures, making direct examination of performance changes across the three time points less precise. It is, however, noteworthy that the pattern of the patient’s performance is similar to performances seen on other tasks of interest in the current study, as well as findings in another study examining short-term verbal memory in adults treated with methylphenidate [37]. Future research in this area should include adequately powered, prospective studies that account for lesion location, neurosurgical history, EEG findings, and varying seizure types when examining the effect of methylphenidate on seizure activity in pediatric patients with epilepsy.

In conclusion, findings from our study highlight the potential for emergence of ADHD after epilepsy surgery and support previous research advocating the measured use of stimulants to treat ADHD patients with epilepsy. Additionally, in our patient we found that post-operative cognitive problems, including attention, executive function, processing speed, and short-term memory may be successfully managed with methylphenidate without seizure aggravation. Although further research is needed to validate these results, it is our hope that more information will emerge as more clinicians consider using methylphenidate to address cognitive symptomatology of ADHD following epilepsy surgery.

## Funding:

The authors received no specific funding for this work.

## Declaration of Competing Interest

The authors declare that they have no known competing financial interests or personal relationships that could have appeared to influence the work reported in this paper.

## Figures and Tables

**Fig. 1a:**
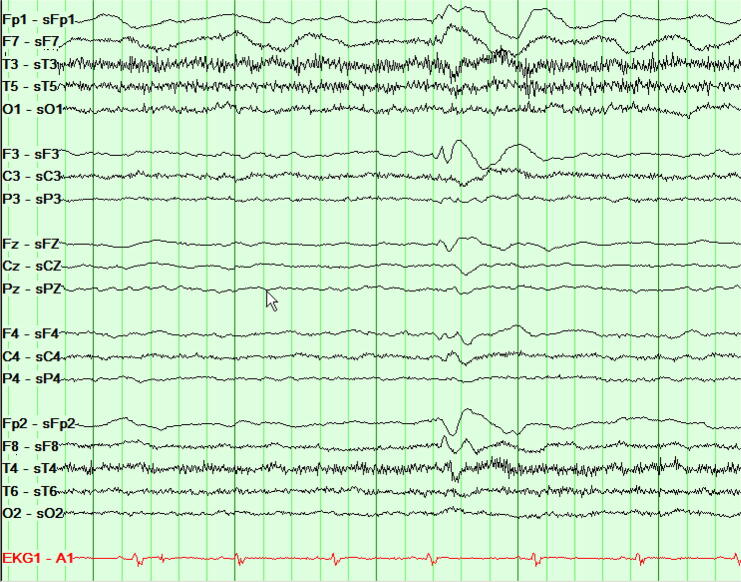
Interictal EEG presented as local reference montage (Laplacian).

**Fig. 1b:**
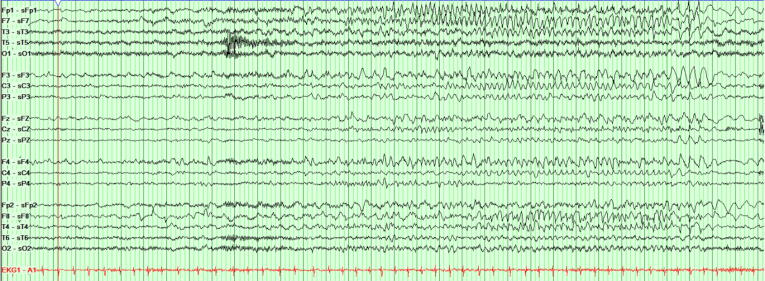
Ictal EEG presented as local reference montage (Laplacian). EEG compressed to demonstrate 33 second epoch (dark green vertical lines represent 1 second).

**Fig. 2a:**
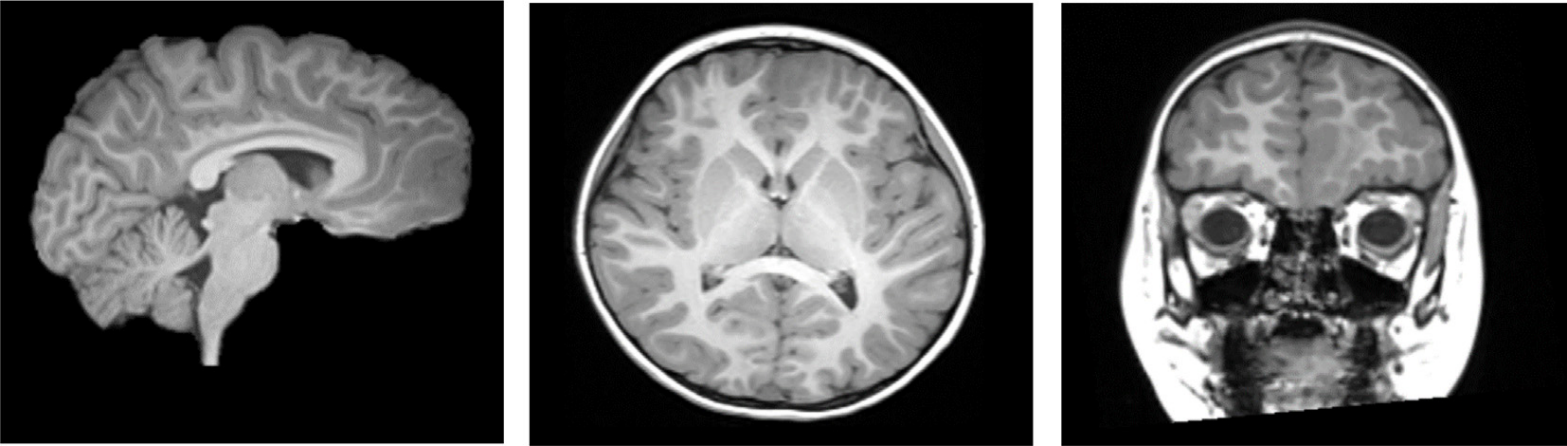
Pre-surgical T1 MRI demonstrating left frontal cortical thickening.

**Fig. 2b:**
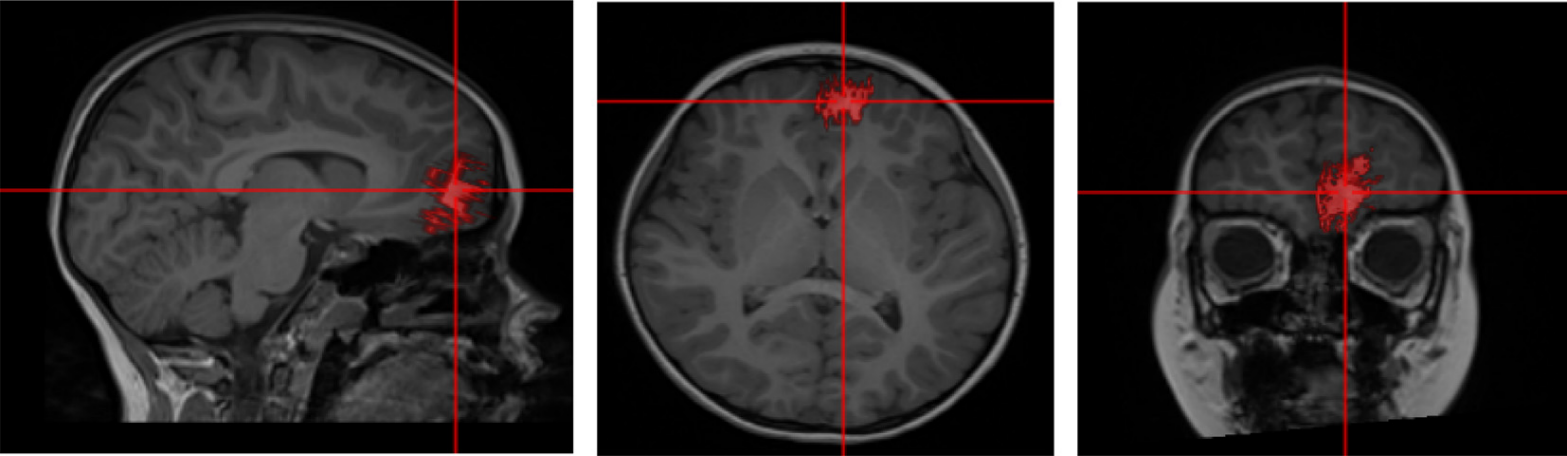
Lesion to be removed highlighted with red markings.

**Fig. 4a:**
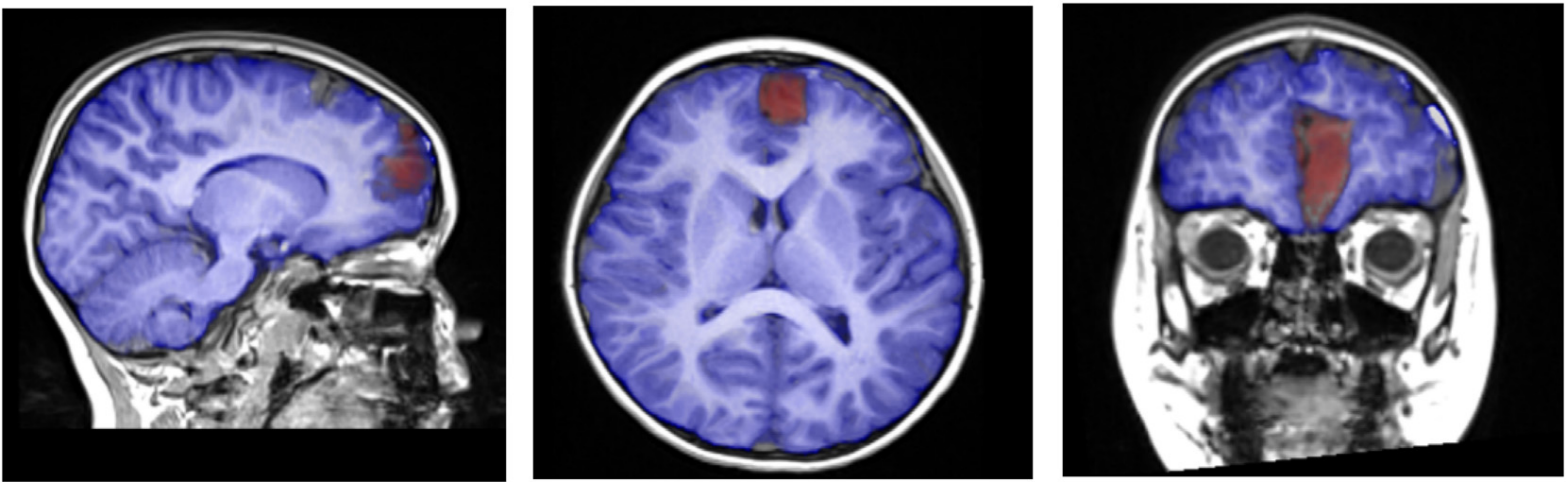
Post-surgery T1 MRI.

**Fig. 4b:**
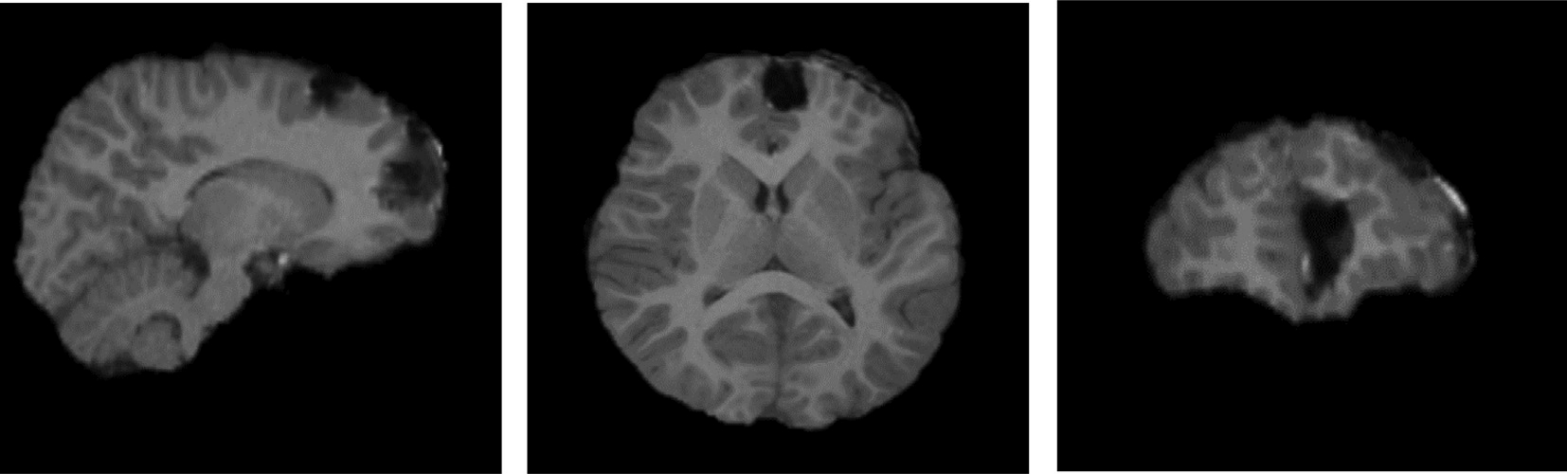
Post-surgery T1 MRI (after skull stripping).

**Table 1:** Standard scores of presurgical neuropsychological evaluation findings in areas of attention, processing speed, short-term verbal memory, and word reading.

Measure	Description	Standard Score
WISC-V Digit Span	Auditory-verbal attention and working memory	85
WRAML-2 Finger Windows	Visual-spatial attention and working memory	90
WISC-V Coding	Processing speed	100
WRAML-2 Story Memory, Immediate	Short-term verbal memory	100
WJ-IV Letter-Word Identification (Form A)	Single-word reading	95

Standard scores (*M* = 100; *SD* = 15); higher scores = better performance.

**Table 2:** T-Scores for attention performance across the three sessions. Performance differences between sessions 1 (unmedicated) and 2 (medicated) are displayed in the first block. Performance differences between sessions 2 (medicated) and 3 (unmedicated) are displayed in the second block.

Conners’ Continuous Performance Test-3	Block 1			Block 2		
	Session 1	Session 2	Difference	Session 2	Session 3	Difference
Response Style						
Detectability	61*	46	>1*SD*	46	56†	1*SD*
Omissions	74**	49	>2*SD*	49	62*	>1*SD*
Commissions	49	41§	*ns*	41§	47	*ns*
Perseverations	59†	44§	>1*SD*	44§	47	*ns*
Hit RT	57‡	51	*ns*	51	59‡	*ns*
Hit RT SD	55†	45	1*SD*	45	52	*ns*
Variability	55†	49	*ns*	49	52	*ns*
Hit RT Block Change	46	49	*ns*	49	57†	*ns*
Hit RT ISI Change	49	49	*ns*	49	53	*ns*

T-Scores (*M* = 50; *SD* = 10); higher scores = poorer performance.

RT: Reaction Time.

*Clinically Elevated; **Very Clinically Elevated; †High Average; ‡A Little Slow; §Low (in occurrence); *ns*: Non-Significant Difference.

*Note*: Score differences of one *SD* or greater were considered mild to moderately significant; score differences of two *SD*s or greater were considered clinically significant.

**Table 3:** Standard scores for processing speed performance across the three sessions. Performance differences between sessions 1 (unmedicated) and 2 (medicated) are displayed in the first block. Performance differences between sessions 2 (medicated) and 3 (unmedicated) are displayed in the second block.

WISC-V Coding	Block 1			Block 2		
	Session 1	Block 1Session 2	Difference	Session 2	Block 2Session 3	Difference
Standard Score	95	105	*ns*	105	90	1*SD*

Standard scores (*M* = 100; *SD* = 15); higher scores = better performance.

*ns*: Non-significant difference.

*Note*: Score differences of one SD or greater were considered mild to moderately significant.

**Table 4:** Standard scores for short-term verbal memory performance across the three sessions.

Measure	Session 1	Session 2	Session 3
WRAML-2 Stories	85	-	-
ChAMP Instructions	-	100	-
CMS Stories	-	-	90

Standard scores (*M* = 100; *SD* = 15); higher scores = better performance

*Note:* Differences in performance across the three sessions could not be quantitatively examined due to use of different measures.

**Table 5:** Standard scores for word reading performance across the three sessions.

WJ-IV Letter-Word Identification	Session 1	Session 2	Session 3
Form A	102	-	-
Form B	-	97	-
Form A	-	-	100

Standard scores (*M* = 100; *SD* = 15); higher scores = better performance.

*Note*: There were no significant differences in word-reading performance across the three time points.
